# Residual Antimalarials in Malaria Patients from Tanzania – Implications on Drug Efficacy Assessment and Spread of Parasite Resistance

**DOI:** 10.1371/journal.pone.0008184

**Published:** 2009-12-14

**Authors:** Eva Maria Hodel, Abdunoor Mulokozi Kabanywanyi, Aggrey Malila, Boris Zanolari, Thomas Mercier, Hans-Peter Beck, Thierry Buclin, Piero Olliaro, Laurent Arthur Decosterd, Blaise Genton

**Affiliations:** 1 Swiss Tropical Institute, Basel, Switzerland; 2 Ifakara Health Institute, Dar es Salaam and Ifakara, Tanzania; 3 Division of Clinical Pharmacology, Department de Medicine, University Hospital and University of Lausanne, Lausanne, Switzerland; 4 UNICEF/UNDP/World Bank/WHO Special Programme for Research and Training in Tropical Diseases (TDR), Geneva, Switzerland; 5 Department of Ambulatory Care and Community Medicine, University of Lausanne, Lausanne, Switzerland; University of Cape Town, South Africa

## Abstract

**Background:**

Repeated antimalarial treatment for febrile episodes and self-treatment are common in malaria-endemic areas. The intake of antimalarials prior to participating in an *in vivo* study may alter treatment outcome and affect the interpretation of both efficacy and safety outcomes. We report the findings from baseline plasma sampling of malaria patients prior to inclusion into an *in vivo* study in Tanzania and discuss the implications of residual concentrations of antimalarials in this setting.

**Methods and Findings:**

In an *in vivo* study conducted in a rural area of Tanzania in 2008, baseline plasma samples from patients reporting no antimalarial intake within the last 28 days were screened for the presence of 14 antimalarials (parent drugs or metabolites) using liquid chromatography-tandem mass spectrometry. Among the 148 patients enrolled, 110 (74.3%) had at least one antimalarial in their plasma: 80 (54.1%) had lumefantrine above the lower limit of calibration (LLC = 4 ng/mL), 7 (4.7%) desbutyl-lumefantrine (4 ng/mL), 77 (52.0%) sulfadoxine (0.5 ng/mL), 15 (10.1%) pyrimethamine (0.5 ng/mL), 16 (10.8%) quinine (2.5 ng/mL) and none chloroquine (2.5 ng/mL).

**Conclusions:**

The proportion of patients with detectable antimalarial drug levels prior to enrollment into the study is worrying. Indeed artemether–lumefantrine was supposed to be available only at government health facilities. Although sulfadoxine–pyrimethamine is only recommended for intermittent preventive treatment in pregnancy (IPTp), it was still widely used in public and private health facilities and sold in drug shops. Self-reporting of previous drug intake is unreliable and thus screening for the presence of antimalarial drug levels should be considered in future *in vivo* studies to allow for accurate assessment of treatment outcome. Furthermore, persisting sub-therapeutic drug levels of antimalarials in a population could promote the spread of drug resistance. The knowledge on drug pressure in a given population is important to monitor standard treatment policy implementation.

## Introduction

The intake of antimalarial drugs prior to inclusion in an *in vivo* study may interfere with the estimation of treatment outcomes (for both efficacy and safety) due to the presence of residual antimalarials. The standard World Health Organization (WHO) protocol for monitoring antimalarial drug efficacy does not exclude patients with a history of previous antimalarial drug use or the presence of antimalarial drugs in the urine or blood [1]. Nonetheless, it is customary in clinical studies to record the occurrence of previous drug intake at screening as reported by the patient, parent or guardian. Two studies in Africa investigated self-reporting of drug intake [2], [3], and both concluded that it is inaccurate. A more objective indication on the drug use in a study population would be obtained by screening the urine or blood for the presence of antimalarial drugs. There are studies on residuals of antimalarials that have been used in past policies, i.e. chloroquine (CQ) or sulfadoxine–pyrimethamine (SP), in urine or blood in the general population or patients [4]–[17]. However, to our knowledge, there is no study on the presence of lumefantrine in malaria patients seeking medical care.

Policy makers in malaria endemic countries are faced with the difficult problem of ensuring easy and early access to effective and high quality antimalarials, while preventing their uncontrolled and unnecessary use, which would increase drug pressure on the parasites and encourage parasite resistance. Thus, knowledge of drug use in a specific area could help decisions makers to assess how treatment policies are implemented.

Here we report the findings of the analysis of baseline samples from patients with *Plasmodium falciparum* malaria recruited in an *in vivo* study in Tanzania. Samples were analysed for the presence of 14 currently in-use antimalarials in a single run using a liquid chromatography-tandem mass spectrometry assay [18].

## Methods

### Ethics Statement

All the applied protocols and related documents were approved by the Ethikkommission beider Basel (EKBB), the Institutional Review Board of the Ifakara Health Institute and the National Institute for Medical Research Review Board. Blood samples were obtained after written informed consent in Swahili from the participants or their responsible guardians.

### Study Area and Population

The study was performed in a rural area with moderate to high malaria transmission intensity (Kilombero district, Morogoro region, Tanzania) during the main rainy season from March to May 2008. At the time of the study, artemether–lumefantrine (AL) had recently been introduced as first-line treatment and was only available at government health facilities to ensure controlled prescription. Before 2006, the official first-line treatment in Tanzania was SP, which had in turn replaced CQ in 2001. In 2008, amodiaquine, SP and quinine were widely available in the private sector in the study area. Artesunate, dihydroartemisinin, and halofantrine could also be found sporadically in a few drug shops (Alba S *et al*., in preparation). In the private sector these drugs could be purchased over the counter without a doctor's prescription.

**Figure 1:**
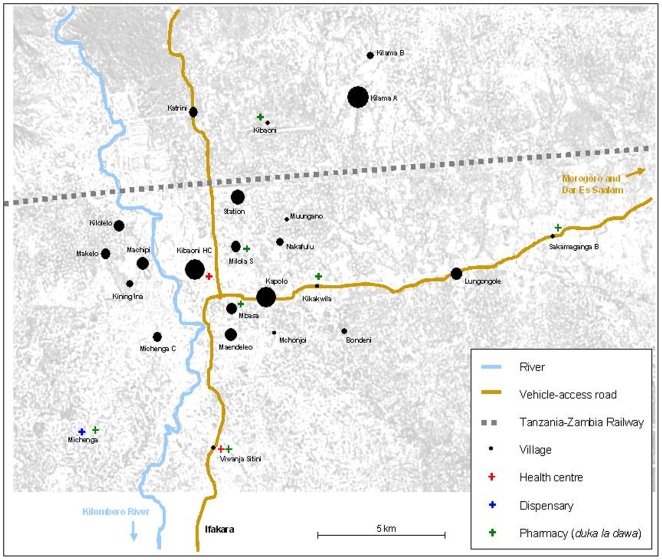
Villages of residence of the patients included in the study.

Febrile patients were recruited at the Kibaoni Health Center, 6 km from Ifakara down town, that serves a population of 26,261. The population lives in villages with good coverage of government health facilities and licensed drug stores (pharmacies, part II drug stores [*duka la dawa baridi*] and Accredited Drug Dispensing Outlets [ADDO; *duka la dawa muhimu*]) [19], [20]. A map of the villages of residence of the patients included in the study with location of health facilities and drug dispensing outlets is presented in Figure 1.

### Clinical Procedures

The trial was designed to assess the effects of the individual pharmacogenetic profiles on the disposition of standard antimalarials (to be reported elsewhere). It was based on the standard WHO protocol for *in vivo* testing. Suspected malaria cases were screened by rapid diagnostic test (Paracheck Pf®, Orchid Biomedical Systems, India) for the presence of *Plasmodium falciparum*. Parasite count and specification of *Plasmodium* were done by microscopy. The patients with a positive result were then seen by a clinical officer, who invited them to participate in the study if they did not present with danger signs of complicated malaria or severe concomitant illness, and if they reported not having taken antimalarials in the previous 28 days. The latter information was checked against the patient's care log book when available. Consenting patients had a baseline sample (Day 0, 4.5 mL venous blood collected in an EDTA Vacutainer®; Becton, Dickinson and Company, USA) taken to check for potential residual antimalarials and correct pharmacokinetic analyses later on. Treatment with the standard first-line treatment AL was then initiated, according to body weight and age category.

### Laboratory Procedures

Blood samples were kept on ice for no longer than 6 h after bleeding (venipuncture) and then aliquoted into whole blood, plasma and pellet and immediately stored at −80°C. Plasma concentrations of 14 antimalarial drugs and their metabolites, i.e. artemether, artesunate, dihydroartemisinin, amodiaquine, *N*-desethyl-amodiaquine, lumefantrine, desbutyl-lumefantrine, piperaquine, pyronaridine, mefloquine, chloroquine, quinine, pyrimethamine and sulfadoxine, were determined by liquid chromatography coupled to tandem triple stage mass spectrometry (LC-MS/MS) [18]. The lower limits of the calibration range (LLC) in our method were selected as the lowest levels of the calibration curves, which confidently provide a bias and CV% below ±20%, in accordance to FDA recommendations [21]. All samples were analyzed twice. First, quantitative measurement was performed using calibration and quality control samples; then, for confirmation, qualitative assessment was repeated using a new chromatographic column that had not been exposed to any antimalarial drugs,. In order to exclude contamination and false positive results, a large set of blank controls was analyzed prior to the clinical samples on the new column, checking for the absence of specific MS/MS signals of the antimalarials investigated.

### Data Management and Analysis

Summary statistics, Chi-square tests, multivariate analysis and graphs of residual plasma concentrations of antimalarials found prior to treatment were produced using Stata® (version 10.1 “intercooled”, Stata Corporation, College Station, TX, USA). Logistic regression analysis was used to investigate the influence of body weight, sex and distance from health facilities or pharmacies on the presence of antimalarials at study entry. The distance between patient home and health facility or pharmacy was defined as “close” or “far” depending on the distance in kilometers, also taking into account ease of access, i.e. main road and river (according to Figure 1). In the first multivariate analysis we considered Bondeni, Kapolo, Katrini, Kibaoni, Kibaoni HC, Kikwawila, Kilolelo, Kining'ina, Machipi, Maendeleo, Makelo, Mbasa, Mchonjoi, Michenga C, Milola S, Muungano, Nakafulu, Sakamaganga B, Station, and Viwanja Stini as “close” and Lungongole, Kilama A, and Kilama B as “far”. In the second analysis we also classified Kilolelo, Kining'ina, Machipi, Makelo, and Michenga C as “far” because the flooding of the Lumemo river might have been an obstacle during the rainy season. We also evaluated the contribution of SP alone, AL alone or both, using likelihood ratio tests.

### Estimation of Time of Drug Intake for Lumefantrine

To estimate the probable timing of drug intake, we compared the plasma concentrations of lumefantrine at baseline (*C_0_*) and on Day 7 (*C_7_*) after a complete treatment with AL for the same patients. We included only patients for whom we had both samples and who complied with the three-day, six-dose AL treatment schedule. Assuming a terminal elimination half-life of *t_½_* = 3.3 days for lumefantrine, an inter-individual variability of 40% [22] and a similar dosage on pre-study exposure and during the study, a back-calculation was done to estimate the intake time before baseline sampling:
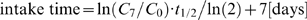



The variability on *t_½_* was used to estimate a 90% confidence interval around this intake time, considering plausible inter-individual variations in elimination rate [22].

### Bayesian Back-Calculations for Sulfadoxine

A Bayesian estimation of the most likely drug intake time was also attempted from individual sulfadoxine plasma concentration data, with a minimization approach using the “Solver” implemented in Microsoft Excel®. Patients were assumed to have taken a single dose of sulfadoxine (combined with pyrimethamine) according to body weight: 1500 mg for >45 kg, 1000 mg for 31–45 kg, 750 mg for 21–30 kg, 500 mg for 11–20 kg and 250 mg for 5–10 kg. Approximate averages of pharmacokinetic parameters, inter-individual variability and intra-individual (residual) variability were derived from a literature review (Tables 1 and 2). These parameters were used to back-calculate the most likely time for dose intake, expected to produce the observed concentration according to a standard one-compartment model. The variability was used to estimate a 90% confidence interval around this intake time, considering plausible variations in clearance, distribution volume and measurement/modeling error [23]. Similar calculations were not attempted for other antimalarials, as their dosage forms are more heterogeneous (lumefantrine and quinine), their population pharmacokinetic parameters are less well characterized (lumefantrine) and their half-lives are shorter (pyrimethamine, quinine).

**Table 1:** Review of pharmacokinetic studies of sulfadoxine.

Study	Number of subjects	Samples per subject	Sex	Age [y]	Body weight [kg]	Condition	Analytic method	Sample type	Dose [mg]	Regimen	PK approach	Compartments
Mansor *et al*. [32]	10	18	males	22–30	51–60	Healthy adults	GC	Plasma	1000	single dose	AUTOAN-NONLIN	2
Obua *et al*. [33]	83	6	both	2.5	11.9±2.7	*Falciparum* malaria	HPLC-UV	Whole blood on filter paper	250 or 500	one dose daily for three days	NONMEM	2
Green *et al*. [34]	33	7	females	15–30	47.2–71.0	*Falciparum* malaria, pregnancy, HIV positiv	HPLC-UV	Whole blood	1500	single dose	log-linear regression	1
Trenque *et al*. [35]	89	3	both	0–14	3–59	Congenital toxoplasmosis	HPLC-UV	Plasma	25/kg	two or three times a month	NONMEM	1
Barnes *et al*. [36]	290	8	both	14	<103	*Falciparum* malaria	HPLC-MS	Whole blood on filter paper	25/kg, ≤1500	single dose	WinNonlin	1
Edstein *et al*. [34]	7		both	Adults		Healthy adults		Whole blood	500	single dose		
Corvaisier *et al*. [38]	32	5	both	0.2–2.1	5–13.4	Congenital toxoplasmosis	HPLC-UV	Plasma	25/kg	one dose every 10 days	NPEM	1
Obua *et al*. [39]	9	14	both			Healthy adults	HPLC-UV	Plasma	1500	single dose	WinNonlin	1

**Table 2:** Review of pharmacokinetic studies of sulfadoxine (continued).

Study	Volume of distribution (*V*)		Clearance (*CL*)		Absorption rate constant (*k_a_*)	Terminal half-life (*t_½_*)	Additive residual variability	Proportional residual variability	Comment
	[L]	[L/kg]	[mL/h/kg]	[L/h]	[h^−1^]	[h]	[µg/L]		
Mansor *et al*. [32]	30.90^a^			0.084±0.013	1.30±1.10	255±61			Mean±S.D.
Obua *et al*. [33]		Central: 0.13±47%; peripheral: 1.6±0%		0.023±33%	0.30		2.60	27%	Estimate±CV%
Green *et al*. [34]	15.0 (12.1–16.0)	0.24 (0.021–0.27)	1.01 (0.82–1.61)	0.066 (0.048–0.092)		148 (121–193)			Median (IQR)
Trenque *et al*. [35]		2.07±0% for 11 kg		0.0108±16.3% for 11 kg	0.055	139 c	8.70	31%	Estimate±CV%
Barnes *et al*. [36]		0.40 (0.29–0.56)	1.85 (1.15–2.89)			161 (105–218)			Median (IQR)
Edstein *et al*. [34]		0.25±0.03	0.79±0.15						Mean±S.D.
Corvaisier *et al*. [38]		0.393±85%	2.07^b^		1.659±94%	132±40%			Mean±CV%
Obua *et al*. [39]		0.15 (0.12–0.18)	0.39 (0.30–0.56)		0.32 (0.29–0.62)	229(139–272)			Median (range)
Overall		0.25±33%	1±33%		1.5±0%	173	0	30%	

a Derived from clearance and plasma half-life (*t_½_*).

b Derived from clearance and elimination rate constant (*λ*).

c Derived from *V*/*CL* × ln(2).

d Corrected by a factor of (*BW*/11 kg)^0.72^ for other body weight (*BW*) values.

e Corrected by a factor of (*BW*/11 kg)^0..64^ for other body weight (*BW*) values.

## Results

A total of 1672 patients of all age were screened, of whom 389 (23%) had a positive malaria test and 150 were eligible and willing to participate in the *in vivo* study. Two patients (one from the Kibaoni HC area and one from Kining'ina) were excluded from the analyses (venipuncture unfeasible in one patient; treatment initiated before baseline sampling in the other one), leaving 148 patients with a valid baseline sample, of whom 64 (43.2%) were male and 84 (56.8%) female (3 (2.0%) pregnant in 3^rd^ trimester). Patients' ages ranged from 1 to 78 years (median 9 years). 51 (34.5%) patients were children under the age of 5, and 94 (63.5%) were <12 years old.

**Figure 2:**
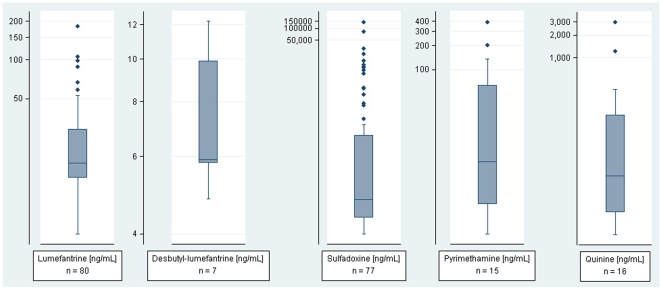
Residual plasma concentrations of antimalarials found prior to treatment in 148 malaria patients. Number of patients (n), median, 25^th^ and 75^th^ percentile, lower and upper adjacent values, and outside values are shown for lumefantrine, desbutyl-lumefantrine, sulfadoxine, pyrimethamine and quinine on a logarithmic scale [ng/mL].

The presence of antimalarial drug was detected in the plasma of 111 (74.3%) patients: 80 (54.1%) had lumefantrine above the lower limit of calibration (LLC = 4 ng/mL), 7 (4.7%) desbutyl-lumefantrine (LLC = 4 ng/mL), 77 (52.0%) sulfadoxine (LLC = 0.5 ng/mL), 15 (10.1%) pyrimethamine (LLC = 0.5 ng/mL), 16 (10.8%) quinine (LLC = 2.5 ng/mL) and none chloroquine (LLC = 2.5 ng/mL) or any other antimalarials tested. Summary statistics are shown in Table 3, and box plots of residual plasma concentrations are represented in Figure 2. Among the 111 patients with residual drug concentrations, 57 (38.5%) had more than one drug (note that parent drug and metabolite or combined regimens such as SP are considered as one): 43 (29.1%) had both lumefantrine and SP, 6 (4.1%) had both lumefantrine and quinine, 1 (0.7%) had both SP and quinine, and 7 (4.7%) had all three agents. The presence of residual antimalarials in plasma was significantly more frequent among children under 5 years of age (86.3%, *χ* ^2^ = 5.82, *P* = 0.016) than among older children and adults (68.0% altogether).

**Table 3:** Residual plasma concentrations of antimalarials found prior to treatment in 148 malaria patients [ng/mL].

Antimalarial	Patients (%)	Mean	Median	Minimum	Maximum
Lumefantrine	80 (54.1)	25.3	15.8	4.4	181.8
Desbutyl-lumefantrine	7 (4.7)	7.3	5.9	4.8	12.2
Sulfadoxine	77 (52.0)	4′480.2	4.4	0.6	138′887.5
Pyrimethamine	15 (10.1)	56.8	7.1	0.9	391.3
Quinine	16 (10.8)	318.0	26.3	4.4	2′947.2

No artemether, artesunate, dihydroartemisinin, amodiaquine, *N*-desethyl-amodiaquine, piperaquine, pyronaridine, mefloquine, or chloroquine was found.

For lumefantrine, among the 59 eligible patients the median plasma concentration (range) was 18.3 ng/mL (4.4–181.8 ng/mL) on Day 0 and 413 ng/mL (37.3–1402 ng/mL) on Day 7. This means that, to account for the levels observed on Day 0, a similar dosage level should have been administered at a median of 21 days (interquartile range 17–24 days, whole range 11–29 days) before study entry. In 2 patients (3%) this estimate was >28 days. The variability in *t_½_* translates into 90% confidence intervals extending from 74% to 144% of estimates (median).

Back-estimation of the most likely times for sulfadoxine intake indicated a median of 108 days before blood sampling at study entry (interquartile range 67 to 121 days, whole range <1 to 130 days). Two patients had concentrations >78 mg/mL, compatible with same day intake. In 70 patients (91%), the estimate exceeded 28 days. The evaluation of uncertainty around individual dose intake times showed 90% confidence intervals extending from 49% to 202% of estimates (median).

The investigation of the influence of body weight, sex and distance to health facilities or pharmacies on the probability of residual antimalarials at study entry (details not presented) showed only a significant relationship between body weight and residual AL levels (likelihood ratio *χ* ^2^ = 9.06, *P* = 0.03 in the first analysis; likelihood ratio *χ* ^2^ = 9.60, *P* = 0.02 in the second analysis), patients with lower body weight being more likely to show residual AL levels. However, this was not the case for SP or SP and AL taken together. Furthermore, neither sex nor distance from health facilities or pharmacies showed a significant effect on residual levels of SP, AL or both at study entry.

## Discussion

This is the first study investigating the presence of a range of antimalarials in the plasma of African malaria patients on enrollment into an *in vivo* study. The measurement of 14 antimalarial drugs currently in-use allowed a comprehensive assessment of drugs available in the community under study.

### Artemether–Lumefantrine

Three in four patients had detectable plasma concentrations of antimalarials at the time of enrolment into the study, and in a majority of cases the agent detected was lumefantrine/desethyl-lumefantrine – indicating that they had taken AL, which was supposedly available only at government health facilities to ensure controlled prescription of first-line treatments. Assuming that the patients had taken a three-day, six-dose AL treatment regimen, most patients (97%) must have taken the drug within 28 days prior to treatment. Furthermore, it is also possible that patients might have taken a sub-therapeutic dose of AL more recently. However, as indicated by the wide variability in elimination half-lives, these values represent only rough estimates. Nevertheless, these findings suggest that at least half of the patients included into our study had taken AL, mostly within the previous month (one month corresponds roughly to the limit of evaluation of past exposure, considering assay LLC for lumefantrine). The Day 7 values observed in this study are comparable with those of a study in Thai patients [median plasma concentration (range) of lumefantrine was 528 ng/mL (49–5175 ng/mL) after 6 doses of AL over 60 h according to body weight [24]] and in patients from Bangladesh [860.3 ng/mL (53.8–6215.0 ng/mL) [25]]. Due to the short half-life of the artemisinin component, e.g. 45 min for dihydroartemisinin, it is likely that none of the patients had taken a co-formulated ACT (e.g AL) within the last 24 hours [26].

### Sulfadoxine–Pyrimethamine

SP was still found in approximately half of the patients, although it had been officially abandoned as first-line treatment since 2006. Assuming that the patients with residual SP in their plasma had taken a single dose of sulfadoxine according to body weight, most patients must have taken the drug ∼3.5 months (median 108 days) prior blood withdrawal. However, 2 patients had sulfadoxine plasma concentrations indicating very recent exposure, and another 8 exposure during the last 4 weeks. Furthermore, it is also possible that patients might have taken a sub-therapeutic dose of SP more recently. These estimates are approximate, as indicated by the wide confidence interval explained by the fair degree of inter-individual variability in clearance, volume of distribution and residual error. The LC-MS/MS method used for the determination of sulfadoxine (LLC = 0.5 ng/mL) would theoretically make it possible to detect traces of sulfadoxine up to 4 months (127 days) after a single dose of 25 mg/kg of sulfadoxine. These findings are sufficient to conclude that a significant number of patients had taken SP for a previous febrile episode, which is against the standard treatment recommendations. Recent surveys on availability of antimalarials have shown that sick people were getting SP from drugs shops, public and private health facilities (Alba S *et al*., in preparation), especially so when clinicians were doubtful about the diagnosis of malaria.

### Chloroquine and Quinine

This study tends to confirm that CQ, replaced by SP as first-line treatment in 2001, has been effectively withdrawn. On the other hand, one tenth of the patients were found with quinine in their plasma. Quinine has an elimination half-life of 16–18 h in malaria patients [27]. After a treatment with 10 mg/kg of quinine dihydrochloride administered 8-hourly orally for 7 days, the plasma quinine levels had fallen to below 0.4 µg/mL in almost all patients 40 h after the last dose on Day 7 [28]. Thus, we infer that most patients with quinine levels in our study must have taken the last quinine dose not more than 2 days before reporting at the health facility.

### High Numbers of Patients with Residual Antimalarials

Why was the number of patients with residual antimalarials so high? Through the demographic surveillance system (DSS) data and a treatment seeking survey in the Ifakara area it was found that approximately 8% of children had fever in the previous 2 weeks when seen between January and April 2008 (Alba S *et al*., personal communication). An epidemiological study which used the Explanatory Model Interview Catalogue (EMIC) reported that 87.5% (78.2–93.8%) of all fevers in children in our study area were treated with one of the recommended antimalarials (at that time SP, amodiaquine or quinine) [13]. Based on these data, one would expect 14% of children to have had an antimalarial treatment in the preceding month (i.e. (0.08×2)×0.875), which is much lower than the proportion of patients with residual antimalarial plasma levels in this study (86.3% for under 5 s, 68% for older children and adults). This large discrepancy could be either due to poor recall of the study subjects in the epidemiological survey or a selection bias. The latter could have arisen either because we captured (i) only patients preferentially seeking antimalarial treatment at a health facility instead at drug shops (∼76.3% of children under the age of 5 years receiving treatment according to the epidemiological study), (ii) patients who are more susceptible to repeated infections (and hence repeated treatments) or are more exposed to infection, or (iii) patients with easy access to treatment. However, similar results were found in two study sites in rural Cambodia (Hodel *et al.*, in preparation).

### Reliability of Medical History

Whatever the reason is for the large number of malaria patients with antimalarials in their blood at study baseline, the fact remains that these patients are the usual subjects investigated in *in vivo* studies and clinical trials. All patients included in the study reported not having taken antimalarials in the previous 28 days. Entry criteria based on self-reporting of previous drug intake (poor recall) or information recorded in the care log book (self-treatment not documented) are thus unreliable at least in this population and for lumefantrine.

### Potential Bias in Drug Safety and Efficacy Assessment

Previous drug intake may affect the current treatment in several ways. Higher drug exposure resulting from cumulative levels may lead to better efficacy or more toxicity. The parasites causing the disease at the time of enrolment may be of a less sensitive population selected by the previous treatment. Thus, previous antimalarial intake may impact on the outcome of the treatment under investigation, and this study shows that only baseline drug concentration measurement in the blood can reliably be used to account for this effect. Our LC-MS/MS assay covers 14 antimalarials in a single run. We can confidently exclude a lack of specificity and false positives as we included blank plasma samples as negative controls and systematically repeated the measurement on a new chromatographic column. Furthermore, we have demonstrated that all drugs are stable for up to 48 h in plasma stored at 4°C [18]. Therefore, even in settings, where no LC-MS/MS instrument is available, samples can be collected in the field, and easily kept and transported to the nearest laboratory where they can be frozen and stored until assayed or further shipped to their final destination. The identification of patients with residual antimalarials allows distinguishing pre-treated from untreated subjects in a secondary analysis. This distinction should probably not aim at excluding pre-treated patients from enrollment in efficacy studies, but rather at addressing the question wether residual antimalarials affect subsequent treatment or not. Detractors may point out that the distinction between pre-treated and untreated patients (i) does not reflect clinical reality and (ii) will increase the number of participants required to maintain statistical power. We believe it could be an important information if some safety or efficacy issues arise from the observations.

### High Drug Pressure as Risk Factor for the Spread of Drug Resistance

There is abundant literature on the effects of inadequate antimalarial treatment on the emergence and spread of resistance. Here, we do not know if the residual drug levels found were from a full or incomplete treatment, if the person had parasites at the time of the previous drug intake, and whether the parasites causing the current episode are the same or a new infection. Be it as it may, the residual levels were not enough to control parasite replication and clinical symptoms. This means that these parasites have been exposed to inadequate drug levels for some time. The chances of drug resistant parasites to be selected depends on several factors, and is higher for patients with no immunity (e.g. young children), drugs with long residence times and resistance being conferred through single point mutations, and for infections with a large parasite biomass [29]. These patients had a mean baseline parasite biomass of ∼9×10^10^ (ranging from ∼1×10^8^ to ∼6×10^11^, data not shown), values which are in line with those reported for symptomatic cases in malaria-endemic areas [30], and were exposed to drug concentrations which were likely to be in the selective window [31].

The findings of this study must be confirmed in other settings as they have potential implications for both clinical research and surveillance (treatment efficacy and safety outcome) and control (pharmaco-epidemiology, adherence to policy).
